# Effectiveness of Treatments That Alter Metabolomics in Cancer Patients—A Systematic Review

**DOI:** 10.3390/cancers15174297

**Published:** 2023-08-28

**Authors:** Santiago Navarro Ledesma, Dina Hamed-Hamed, Ana González-Muñoz, Leo Pruimboom

**Affiliations:** 1 Department of Physiotherapy, Faculty of Health Sciences, Campus of Melilla, University of Granada, Querol Street 5, 52004 Melilla, Spain; dhamed@correo.ugr.es (D.H.-H.); agonzalezm@correo.ugr.es (A.G.-M.); 2 Department of Physiotherapy, University Chair in Clinical Psychoneuroimmunology, University of Granada and PNI Europe, 52004 Melilla, Spain; leo@cpnieurope.com

**Keywords:** metabolomics, metabolome, genetics, mycobiome, microbiota, neoplasms, cancer pain, pain and quality of life, oncometabolite

## Abstract

**Simple Summary:**

This review is the first study that has identified the metabolomic changes in different types of cancer and the use of metabolomic-based interventions in those patients. Personalized medicine interventions based on metabolomics profiles are proposed for those suffering from different types of cancer. Characteristic metabolomics and personalized interventions and the potential benefits of researching metabolism in cancer are discussed.

**Abstract:**

Introduction: Cancer is the leading cause of death worldwide, with the most frequent being breast cancer in women, prostate cancer in men and colon cancer in both sexes. The use of metabolomics to find new biomarkers can provide knowledge about possible interventions based on the presence of oncometabolites in different cancer types. Objectives: The primary purpose of this review is to analyze the characteristic metabolome of three of the most frequent cancer types. We further want to identify the existence and success rate of metabolomics-based intervention in patients suffering from those cancer types. Our conclusions are based on the analysis of the methodological quality of the studies. Methods: We searched for studies that investigated the metabolomic characteristics in patients suffering from breast cancer, prostate cancer or colon cancer in clinical trials. The data were analyzed, as well as the effects of specific interventions based on identified metabolomics and one or more oncometabolites. The used databases were PubMed, Virtual Health Library, Web of Science, EBSCO and Cochrane Library. Only nine studies met the selection criteria. Study bias was analyzed using the Cochrane risk of bias tool. This systematic review protocol was registered at the International Prospective Register of Systematic Reviews (PROSPERO: CRD42023401474). Results: Only nine studies about clinical trials were included in this review and show a moderate quality of evidence. Metabolomics-based interventions related with disease outcome were conflictive with no or small changes in the metabolic characteristics of the different cancer types. Conclusions: This systematic review shows some interesting results related with metabolomics-based interventions and their effects on changes in certain cancer oncometabolites. The small number of studies we identified which fulfilled our inclusion criteria in this systematic review does not allow us to draw definitive conclusions. Nevertheless, some results can be considered as promising although further research is needed. That research must focus not only on the presence of possible oncometabolites but also on possible metabolomics-based interventions and their influence on the outcome in patients suffering from breast cancer, prostate cancer or colon cancer.

## 1. Introduction

Cancer, a public health problem [1], is one of the main causes of mortality and morbidity [2]. In Spain, nine million people are diagnosed with cancer annually [3] making it the second leading cause of death in Spain [2], whereas it is the leading cause of death worldwide [4]. Cancer is a multifactorial illness, produced by environmental, occupational, social, and lifestyle factors and their interaction with genetics and the cell metabolism [2]. Often less recognized, the consideration of psychological and social aspects as possible risk factors and their comprehensive and multidisciplinary management is becoming increasingly important [5]. Contemporary cancer treatment is still very much based on the use of chemotherapeutic agents and more recently on immune modulators [4]. The success of these therapies is mostly related to an increased life expectancy but often with the burden on the quality of life [3]. Current therapies are mostly ‘one target’ interventions that underestimate the complexity of cancer as a systemic disease caused by long-term irritating lifestyle factors leading to a state of low-grade inflammation, also possibly caused by the aging process and possible (sub) clinical infections, opposite to the still prevailing opinion that cancer is a genetic disease [6].

Lifestyle factors such as an inadequate diet over a prolonged period, excessive alcohol consumption, physical inactivity, and being overweight seem to produce more than 20% of most cancer cases and therefore cancer prevention programs should include lifestyle modifications [7]. For example, frequent engagement in physical activity can already strongly reduce the risk of developing breast, colon, endometrial, bladder, stomach, esophageal, kidney, and prostate cancer [8] and many of them are the most frequent ones [9].

Wishart [10] comprehensibly describes the impact of modern life risk factors and genetics on the development of multiple cancer types. The outcome, not surprisingly, is that 73–80% of all cancer types are caused by lifestyle, pollution, and other environmental factors and breast, prostate and colon cancer, the three cancer types investigated in this review, are no exceptions.

Knowing that metabolomics as a science is still very young, we opted to investigate metabolomics for the three most-frequent cancer types, as we expected that the study number would be low. The result sector shows that we only could identify a few studies even in these three most frequent cancer types (breast, prostate and colon).

The use of metabolomics as one of four-omics (genomics, transcriptomics, proteomics) in cancer research, affords the possibility to understand complex biological systems at the root of the development of cancer and identify the cellular phenotype belonging to the different types of cancer [11]. The plus value of the use of metabolomics in cancer is the fact that it provides a direct read-out of the phenotype related to the specific cell type in different types of cancer. Metabolomics, when used in the right way, shows the sum of distortions at protein, DNA, RNA levels and the way cells have altered their metabolism providing knowledge about specific oncometabolites [12,13].

As cancer is considered more and more a metabolic disease caused by multiple risk factors as part of the actual Anthropocene, it seems logical to include the science of metabolomics in detecting new targets for the treatment and prevention of cancer in more- or less-susceptible people, which is the focus of our systematic review. Never have humans been exposed to so many ‘new’ challenges, such as multiple toxic chemicals, food abundancy, sitting time, and many others. It seems logical that all those new risk factors affect genetics and immunological, endocrinological and metabolic pathways, measurable with the science of metabolomics [11,14,15].

Some studies already mention the possible oncometabolites related to the development of the three most frequent cancer types, being breast, colon and prostate cancer.

Polyamines play a possible role as oncometabolites in breast cancer. Breast cancer is the most common malignancy among women worldwide [16,17]. Early detection and advances in cancer treatment have attested to a 10-year survival rate in 80% of women with breast cancer [17]. Polyamines have been associated with rapid tumor growth through biosynthesis and accumulation in different tissues. The accumulation of polyamines has been evidenced by increased plasma and urine concentrations of polyamines such as spermine and spermidine in breast cancer patients [18,19].

In prostate cancer, PSA (prostate-specific antigen) remains the most investigated protein. Prostate cancer is more common in men over 70 years of age [20], with aging being the most significant risk factor [21]. The development of prostate cancer is a complex and systemic process and scientific evidence indicates that multiple exogenous factors affect the progression of the disease [22], causing prostate cancer in one of every eight men worldwide [21]. The incidence of men suffering from prostate cancer still increases in most developed countries [23] whereas a trend of increased life expectancy is also observed [24]. A recent study [25] quantifies the impact of several risk factors on the development of prostate cancer in 41830 European Americans (EAs) and 1282 African Americans (AAs) as part of the Prostate, Lung, Colorectal, and Ovarian Cancer Screening Trial (PLCO) project. The outcome was that the top six risk factors rank as follows, using PSA as primary biomarker [25]: age > PCa-fh (family history of prostate cancer) > diabetes ≥ race > lifestyle (smoking and coffee consumption) ≥ marital status ≥ BMI > X, in which X represented a specific diet nutrient/ingredient metric.

Other different and very useful factors in the development of interventions regarding oncometabolites related with prostate cancer are choline [26], glucose [10], nitric oxide [27] and uric acid [28]. Although these oncometabolites seem related with the severity and mortality in patients suffering from prostate cancer, interventions reducing the presence of for instance uric acid do not show a significant reduction in tumor growth or overall survival [29]. These results show the difficulty in understanding the way cancer cells use oncometabolites to proliferate and progress, making the interpretation of metabolomics essential.

Although new oncometabolites have been found in patients suffering from prostate cancer, PSA, as a specific oncometabolite, is still used as the primary biomarker for the detection and diagnosis of prostate cancer [22].

Colorectal cancer is the third most-prevalent cancer and one of the leading causes of death worldwide [14,30]. Despite this, the survival rate is high if it is detected and treated in its early stages [31]. The incidence of colorectal cancer increases with age, with most cases being diagnosed after age 50. Ninety per cent of cases are considered sporadic (non-hereditary) and the rest may be hereditary [32]. The most common symptoms of this type of cancer are changes in bowel habits and the appearance of blood in the stool [32]. Different oncometabolites in colon cancer have been identified including methionine [33], which is a promising substance because of the possibility to intervene with a methionine and tryptophan depletion diet in patients suffering from colon cancer [33].

Many studies have confirmed that an inadequate diet and unhealthy lifestyle play an important role in the development of colorectal cancer and high methionine intake through a red meat-rich diet is an important risk factor for colon cancer [34].

Colon cancer progression seems to be related with other oncometabolites that are part of the acyl-CoA synthetase/stearoyl-CoA desaturase (ACSL/SCD) lipid network [15].

Metabolomics is part of omics sciences, [35] and has been used for the discovery of tumor biomarkers in recent years [30]. It can be considered as an accurate, coherent, and quantitative method for examining multiple mechanisms related to cell growth, metabolism, apoptosis and possible cancer development to compare those outcomes with normal functioning of cells [36]. Metabolomics measurements are performed in urine, serum and less frequently in fecal extracts, saliva and amniotic fluid [36]. Due to the high sensitivity of this technique, unusual changes in the metabolome can be identified at an early stage of many diseases, including cancer, and allow for early diagnosis [37]. The actual state-of-the-art related to the science of metabolomics justifies its use as a method for identifying new cancer biomarkers and oncometabolites, and the subsequent development of cancer-specific oncometabolite-directed interventions and personalized medicine [38,39]. The aim of our systematic review is to add knowledge to the metabolomics of the most-frequent cancer types and to offer new treatment options for people suffering from this devastating disease.

## 2. Materials and Methods

### 2.1. Study Design

A systematic review was conducted following the recommendations of the Preferred Reporting Items for Systematic Review and Meta-Analysis (PRISMA) [40], which includes only randomized controlled trials. The process was carried out using the PICOS strategy. The protocol for this review was registered in the International Register of Systematic Reviews (Prosperous CRD42023401474). The purpose of our review and study was to find scientific evidence on breast, prostate and colon cancer metabolomics, detect possible interventions that directly influence essential oncometabolites and the outcome of disease, using those interventions.

### 2.2. Documentary Sources Consulted

Sources used for the manuscript search were PubMed, Scopus, Virtual Health Library (VHL), Cochrane Library and the Web of Science.

### 2.3. Search Strategy

Keywords used and extracted from thesaurus Medical Subject Headings (MesH) were: “Metabolomics”, “metabolome”, “genetics”, “mycobiome”, “microbiota”, “neoplasms”, “cancer pain”, “pain” and “quality of life”. The following non-MesH thesaurus terms were also used: metabolites, human genetics and microbiome. The terms were combined with the Boolean operators AND and OR. The terms had to appear in the title, abstract and keyword list.

The last search was conducted on 10 February 2023.

In the Supplementary Materials, Table S1 shows the search strategies that were used for the detailed studies.

### 2.4. Inclusion Criteria

The inclusion criteria were as follows:

− Human randomized controlled clinical trials published between 2016 and 2023.
− The use of English or Spanish language.
− Breast cancer, colon cancer and prostate cancer.

### 2.5. Exclusion Criteria

All types of cancer other than breast cancer, prostate cancer or colon cancer were excluded.

### 2.6. Study Selection Process

The Rayyan QCRI program [41] was used for the storage and subsequent removal of duplicates of the included studies. The process of selection and identification of the studies was carried out by means of selective reading of the title and the abstract. Subsequently, a full-text reading of the articles that apparently met the inclusion criteria was carried out by all authors of this systematic review.

### 2.7. Data Extraction

The PICOS strategy was used for data extraction and included the following data characteristics: author, year of publication, place where the study was conducted and the type of cancer. Additionally, data were also extracted on sample characteristics (size, age, sex), characteristics of the intervention (type of intervention, duration, metabolomics, and changes in metabolites) and main outcomes (assessment tools; follow-up and intervention outcomes).

### 2.8. Risk of Bias Measurement Tool

The risk of bias tool proposed by the Cochrane Manual of Systematic Reviews of Interventions was used to assess the risk of bias of included studies [42]. This tool assesses seven domains, where each domain is evaluated with three possibilities: “high risk” (−), “low risk” (+) and “unclear risk” (?). The domains that are used for the detection risk of bias are: selection bias, performance bias, detection bias, attrition bias, reporting biases and finally other sources of bias. All these domains help to qualify the level of scientific evidence of the included studies.

### 2.9. Quality of the Evidence

The Grading of Recommendations, Assessments, Development and Evaluation (GRADE) tool [43] was used to assess the quality of the evidence for the results of the included studies. This system defines the quality of the evidence as the degree of confidence and the possibility to estimate if a certain effect is significant enough to make a clinical recommendation. Assessment of the quality of evidence includes the risk of bias, inconsistency, imprecision, publication bias, indirect results and other factors.

## 3. Results

### 3.1. Study Identification and Selection Process

In the process of identifying and selecting articles, a total of 6854 articles were located in the different computerized databases. After the elimination of duplicates, the title and abstract of 44 articles were read to assess whether the selected articles met the inclusion criteria. A total of nine articles met these criteria and the full texts were evaluated.

**Figure 1:**
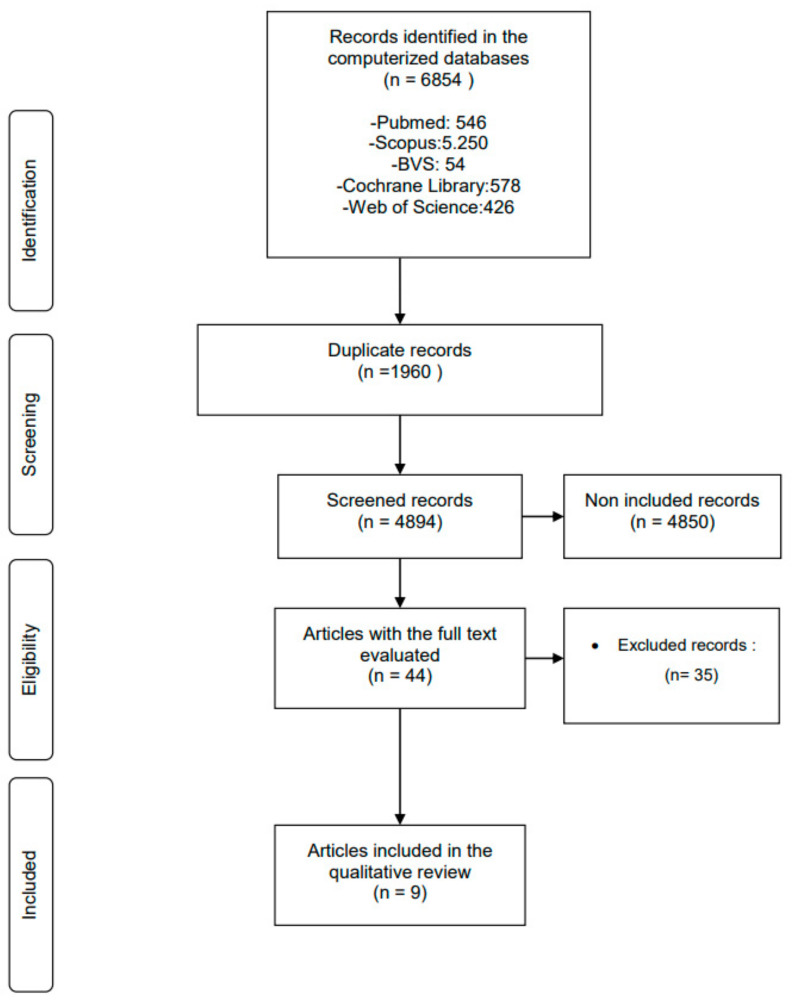
Flow diagram illustrating the study process.

Finally, after full text reading, the nine previously identified studies [44,45,46,47,48,49,50,51,52] were included in this systematic review and a flow diagram of the search strategy was developed (Figure 1).

### 3.2. General Characteristics of the Selected Studies

The studies included in this systematic review were randomized controlled studies as a basic condition of this systematic review [44,45,46,47,48,49,50,51,52]. The publication period for these nine studies spanned from 2019 to 2021, with 2021 [46,47,48,49] being the year with the highest number of the included articles. Most included studies were published very recently, and this highlights the use of metabolomics as a contemporary science in medicine.

Of the nine studies, two were conducted in the United States [46,51], two in Spain [45,52], one in France [49], one in Italy [44], one in China [47], one in Japan [50] and the remaining article in Switzerland [48].

The sum of the sample size of the nine included studies brings together a total of 280 individuals. There were no adverse effects reported related with the interventions that were used to target several oncometabolites found in different types of cancer.

In relation to gender, the study population is made up of men and women. The patients in the studies, who suffer from breast cancer, prostate cancer or colon cancer, are all 45 years old or older.

The following table (Table 1) shows the characteristics mentioned in the studies.

### 3.3. Risk of Bias in the Included Studies

The assessment of risk of bias in all the articles in the studies was high in most fields.

Blinding of participants and personnel and the controlled blinding of evaluators indicated a high risk of bias in the articles by Pietri et al. and Lee et al. [44,48].

Table 2 shows the risk of bias of the included studies. The different colors that appear in the table present the methodological quality of the studies: unclear risk (yellow) and low risk of bias (green).

### 3.4. Intervention Characteristics

All the studies show a study design including an intervention and a control group to investigate the response on an oncometabolite targeting intervention in patients suffering from breast, colon or prostate cancer. In three studies [44,47,48] the intervention was pharmacological, while in one study the effects of a medicinal herb were studied in people suffering from colon cancer [50], whereas another study investigated the impact of an aerobic exercise program in women with breast cancer [49] and finally the remaining four studies used different dietary strategies [45,46,51,52].

All studies researched the impact of the intervention on different oncometabolites through metabolics testing in plasma or urine before and after the intervention.

(1) Pietri et al. [44] studied the effects of DHEA intake, 100 mg/day orally on post-menopausal patients with breast cancer with the main outcome safety. The duration of treatment was 8 weeks and an intervention and a control group were included.
(2) Ávila-Gálvez A et al. [45] studied the impact of a multi-nutrient supplement on women with breast cancer after biopsy-confirmed diagnosis for surgery. Nineteen breast cancer patients consumed three capsules daily whereas the control group (n = 8) did not receive any additional treatment. The multi-nutrient capsules contained pomegranate, orange, lemon, olive extracts, cocoa and grape seed.
(3) Chi et al. [46] studied the metabolomic changes in a low-carbohydrate diet in conjunction with androgen deprivation therapy. Fasting blood samples were taken for the control of glucose, insulin, protein C, lipids, etc., and these samples were used for metabolomic analysis. Eleven participants in the intervention group finalized the study (11/20), whereas 18 in the control group also finalized the study (18/20).
(4) Qu et al. [47] studied the effects of the combined application of neoadjuvant docetaxel and androgen deprivation therapy in people with prostate cancer. The purpose of this study was to investigate, with the use of metabolomics, the difference in endogenous tumor metabolism in prostate cancer patients who received or did not receive neoadjuvant therapy. The cohort consisted of 42 patients receiving the combined neoadjuvant therapy before radical prostatectomy whereas 54 patients in the control group were operated on without additional intervention next to the radical prostatectomy.
(5) Lee et al. [48] studied the metabolic effects of intravenous selenium injections in breast cancer patients. A placebo (n = 14) and an experimental group (n = 15) were included in the study design.
(6) Febvey-Combes et al. [49] studied the effects on metabolomics of an aerobic exercise program in breast cancer patients. A six-month long combined program including aerobic exercise and nutritional changes were added to the current chemotherapy treatment in women (n = 40) with breast cancer, whereas the control group (n = 18) only received chemotherapy.
(7) Hanada et al. [50] studied the effects of a Chinese herbal medicine, Daikenchuto, on the metabolites of patients with colon cancer after a left-sided laparoscopic colectomy. Nine patients received the herbal medicine for 6 months whereas the control group did not receive any additional treatment to the colectomy.
(8) Zarei et al. [51] studied the effects of bean intake on metabolomics measured in plasma and urine of obese and overweight colorectal cancer survivors. Plasma and urine samples were collected at baseline, 2 weeks and 4 weeks after consumption. The study included an intervention group and a placebo meal-receiving group of in total 20 participants.
(9) Ávila-Gálvez B et al. [52] studied the presence of isoflavones, curcuminoids and lignans (polyphenols) in the tissue of people with breast cancer after the intake of three capsules daily, containing the aforementioned substances daily. The metabolic profiles of these polyphenols in normal and malignant breast tissue in newly diagnosed breast cancer patients and the anticancer activity of metabolites produced in tissues were evaluated. The patients were randomized into two groups; the patients of the experimental group consumed three capsules daily until the day of surgery. The control group did not receive any type of supplementation before they were operated on.

Table 3 shows the intervention characteristics in detail.

### 3.5. Results of Oncometabolite Targeting Interventions

This systematic review consists of nine randomized controlled trials with great disparity regarding the results of oncometabolite targeting interventions. The results have been divided according to the type of cancer examined and are as follows:

1. Breast cancer: five studies researched metabolomic changes in breast cancer (Table 4).
  (a) Pietri et al. [44]: no clear changes were observed in metabolites during the 8 weeks of treatment; the authors indicate that this may be due to the small sample size.
  (b) Ávila-Gálvez A et al. [45]: some changes were detected in the following oncometabolites after the intervention were urolithin A-3-O-glucuronide, 2,5-dihydroxybenzoic acid and resveratrol-3-O-sulfate.
  (c) Lee et al. [48]: in this study, the levels of corticosterone, LTB4-DMA and PGE3, which are anti-inflammatory compounds, were found to be significantly higher in the experimental group compared with the control group.
  (d) Febvey-Combes et al. [49]: after 6 months of intervention, no metabolomic changes were observed between the subjects who engaged in the experimental group and those in the control group. Inflammatory biomarkers increased slightly in both groups but no significant differences were observed between groups.
  (e) Ávila-Gálvez B et al. [52]: in the experimental group, high concentrations of curcumin were present in mammary tissues. The use of curcumin could offer long-term anticancer effects.
2. Prostate cancer (Table 5):
  (a) The study by Chi et al. [46] in the experimental group comprised a combination of a dietary intervention along with androgen deprivation therapy. Several changes were found in the experimental group such as a decrease in steroid synthesis, and a reduction in androgen levels, which were associated with higher serum glucose levels. In addition, 3-hydroxybutyric acid and ketogenesis decreased, and acyl-carnitines and 3-formyl-indole were reduced with these changes being associated with androgen deprivation therapy.
  (b) The study by Qu et al. [47] investigated a combined neoadjuvant therapy with androgen deprivation therapy (experimental group) versus androgen deprivation therapy only (control group). Nucleotide synthesis, lipids, citric acid, and glutathione metabolism were all beneficially changed after the combined treatment in prostate cancer patients compared with the control group.
3. Colon cancer—colorectal (Table 6):
  (a) Hanada et al. [50]: metabolome and gut microbiome analyses showed that the levels of plasma lipid mediators associated with the pro-inflammatory arachidonic acid cascade were lower in the experimental group than in the control group, which suspects a reduction in inflammatory activity in those patients using Daikenchuto as a complementary intervention.
  (b) Zarei et al. [51]: the following metabolites which all have protective actions against cancer showed an increase in the experimental group only: (i) 2,3-dihydroxy-2-methylbutyrate, (ii) S-methylcysteine and pipecolate in plasma and (iii) S-adenosylhomocysteine and (iv) cysteine in urine. These promising results justify further studies of the effects nutritional interventions in people suffering from colon cancer and a primary intervention study could also be conducted.

### 3.6. Grade System

This systematic review has a moderate evidence quality. Assessments have relied heavily on the trials’ risk of bias and the imprecision of their results.

For more detailed information, see Table S2 in the Supplementary Materials.

## 4. Discussion

The main purpose of this systematic review was to analyze the impact of oncometabolite-targeting interventions on metabolomics in patients suffering from breast, prostate or colon cancer. We found that the studies included in this review showed a moderate quality of evidence and high disparity in the results.

Nine randomized controlled clinical trials [44,45,46,47,48,49,50,51,52] fulfilled our inclusion and exclusion criteria and we organized the results according to the type of cancer.

Nevertheless, a comparison of the different interventions even within the same cancer type is at least doubtful. Studies are so scarce and one-target focused that it has even been predicted that a positive outcome of these interventions would be at most minimal. Therefore, we discuss all studies separately without the intention to compare the results intra- and inter-cancer type.

The study by Pietri et al. [44], based on the use of DHEA as a complementary intervention in people suffering from breast cancer, did not find any clear changes in metabolites related with that type of cancer. The authors state that this could be due to the small sample size of the study. The study by Lee et al. [48], based on intravenous selenium application, did find elevated levels of anti-inflammatory compounds in the experimental group of breast cancer patients compared with the control group. The results of the latter study invite the development of human clinical trials with a larger sample size. Selenium is an important anti-oxidant and could possibly reduce oxidative damage in patients suffering from breast cancer [53,54]. Selenium also shows potent anti-inflammatory activity [55] which could also explain the positive effects on the metabolomics spectrum observed in the experimental group in the study of Lee et al. [48]. Selenium intake and DHEA production are negatively related; a higher selenium intake is related with lower DHEA levels in women suffering from polycystic overia syndrome [56]. The fact that DHEA supplementation did not show any effect in women suffering from breast cancer [44], whereas selenium does [48], invites the question if DHEA as an oncometabolite should be decreased in patients with breast cancer and not increased. The latter possibility only shows how difficult it is to interpret metabolomics and the evidence of its infancy.

Ávila-Galvez et al. [45,52] used dietary strategies as an intervention, with favorable changes in the patients' metabolites being obtained, and those changes offered protective and anti-cancer actions. These findings are consistent with studies by Kunihiro et al. [57], Bahrami et al. [58] and James et al. [59], who claim that curcumin has anti-proliferative effects in cancer patients. Curcumin has glucose-lowering effects and could therefore be used as complementary treatment to decrease the use of glucose as a precursor of multiple oncometabolites produced by tumor cells [60]. In the study by Febvey-Combes et al. [49], chemotherapy is combined with a 6-month combined program of aerobic exercise and nutrition. No significant metabolomic changes were found in the experimental group with respect to the control group. However, this is opposite to a study by Schmidt et al. [61] that showed that breast cancer patients had a significantly higher level of pro-inflammatory cytokines after undergoing radiotherapy treatment. This effect was counteracted by resistance exercise training, with lower interleukin-6 (IL6) and interleukin-1 (IL1) levels seeming to calibrate the beneficial effect of exercise in these patients. [59]. A meta-analysis on head and neck cancer also found positive effects of using exercise as an additional cancer treatment [62].

Androgen deprivation therapy is the main treatment strategy for men with prostate cancer. The study by Chi et al. [46] added a low-carbohydrate diet intervention to the current androgen deprivation therapy, which favored an anti-inflammatory effect in the experimental group. Androgen deprivation therapy can cause negative effects on several tissues causing metabolic abnormalities. A meta-analysis [63] has proposed healthy dietary interventions to moderate the side effects of androgen deprivation therapy and the results of the study of Chi [46] confirms the possible positive effect of complementary diet changes in the treatment of individuals suffering from prostate cancer.

The study by Qu et al. [47] also shows positive effects when neoadjuvant therapy is added to the regular treatment in patients suffering from prostate cancer. This beneficial effect seems to be related to reduced energy metabolism in prostate cancer tissue and through this pathway tumor growth can possibly be inhibited. The study by Zichri et al. [64] analyzes mitochondrial membrane changes in colon and prostate cancer, noting that during cancer there is increased enzymatic activity of cytochrome C oxidase (mitochondrial protein) involved in cellular energy metabolism [64] and the use of a combined neoadjuvant therapy could counter these metabolic changes in prostate cancer tissue. Both identified studies in men suffering from prostate cancer are not comparable in any way. The interventions activate completely separate mechanisms and test different metabolic outcomes.

Finally, the two colon cancer studies. The study by Hanada et al. [50] studied the effectiveness of a Japanese herbal formula called Daikenchuto, which has been used normally as a symptom reducing complementary intervention in patients suffering from colon cancer. The positive effects showed only in the experimental group further justify investigations in clinical studies with a bigger sample size, not only for disease reduction but also to study the mechanisms of action of Daikenchuto, as stated by the authors of this study. Daikenchuto has proven anti-inflammatory activity, increases colonic blood flow and accelerates bowel movements, reducing the transit time of faeces [65].

The study by Zarei et al. [51] investigated white bean intake in colorectal cancer survivors and revealed changes in amino acid and lipid metabolic pathways as a goal that possibly could be responsible for a reduction in colorectal cancer recurrence [40]. These results were also found in an earlier systematic review in which amino acid and lipid metabolism in colorectal cancer were analyzed [66]. Future studies combining both interventions are warranted because both show positive results. Such research could explore the potential for synergistic effects or unexpected outcomes resulting from the combination.

Finally, this is the first systematic review that analyzes certain changes in the metabolomics spectrum in patients suffering from breast, colon or prostate cancer after a single oncometabolite targeting intervention. The studies included in this systematic review show some promising results although there is no consensus between them. Cancer is a complex systemic disease and ‘one-target’ interventions in current oncology show only partial success and it seems that the same holds for single oncometabolite targeting interventions, natural or not. Cancer must be considered systemic and multifactorial which means that successful treatment should probably also be muti-factorial and systemic.

Subsequently, future research is desirable where the methodological quality of metabolomic change research should be improved and with the possibility of finding effectful treatment options based on the promising science of metabolomics. Single-target interventions should be replaced by combined ones. Contemporary research already shows the cumulative effect of combined interventions as the primary preventive treatment option [67].

### 4.1. Practical Application

Although it is not yet clear what the characteristic metabolome of each type of cancer is, interventions that produce favorable changes in the metabolites of these patients have been detected, which could be related to an improvement in the disease. Not only single-target interventions on metabolomics in cancer patients should be investigated but also combined and multi-target interventions influencing multiple mechanisms.

#### Study Limitations

Our study has several limitations. Firstly, the total sample size of all studies together (270) is too low to draw any significant conclusion other than to advise to continue to research treatment options using metabolomics diagnosis. Another limitation is the relative low quality of existing scientific evidence on the study of metabolomic changes that cancer patients may undergo during and after treatment. Nevertheless, this systematic review opens new research options in patients suffering from different types of cancer. As already stated, we propose to investigate multi-target interventions in people suffering from cancer, a complex and systemic disease with devastating effects on the human population.

### 4.2. Prospective

Future studies that clarify the metabolomic profile of cancer patients and their changes after the indicated treatments are necessary to optimize current treatments and propose personalized medical approaches.

## 5. Conclusions

This systematic review proves that the science of metabolomics is still in its late infancy, specifically in cancer research. Although many oncometabolites have been identified in many cancer types, interventions are still scarce. As cancer is mainly a metabolic disease, oncometabolite-based interventions should be successful, nevertheless, how oncometabolites interplay with the disease is not fully understood and therefore metabolomics interpretation must be made with caution. Additionally, the role of methionine in colon cancer should be further studied since it may present a protective action. Further research is needed to determine characteristic biomarkers in different types of cancers and possible new treatment options.

## Figures and Tables

**Table 1:** Characteristics of the included studies.

Author	Year	Country	Cancer	Sample	Gender	Age (Years)
Pietri et al. [44]	2019	Italy	Breast Cancer	N = 18	Female	74
Ávila-Galvez et al. [45]	2019	Spain	Breast Cancer	N = 27	Female 19 Female 8 (CG)	56 ± 10
Chi et al. [46]	2020	USA	Prostate Cancer	N = 40	Male	-
Qu et al. [47]	2021	China	Prostate Cancer	N = 32	Male	64 (57–75)
Lee et al. [48]	2021	Switzerland	Breast Cancer	N = 29	Female	-
Febvey-Combes et al. [49]	2021	France	Breast Cancer	N = 58	Female	53.8
Hanada et al. [50].	2021	Japan	Colon Cancer	N = 8	Male/female 4/4	64.0
				N = 9	Male/Female 6/3	
Zarei et al. [51]	2021	USA	Colorectal Cancer	N = 20	Male Female	-
Ávila-Galvez B et al. [52]	2021	Spain	Breast Cancer	N = 39	Female	55 ± 14

**Table 2:** Risk of bias.

	Pietri et al. [44]	Ávila-Gálvez A et al. [45]	Chi et al. [46]	Qu et al. [47]	Lee et al. [48]	Combes et al. [49]	Hanada et al. [50]	Zarei et al. [51]	Ávila-Gálvez et al. [52]
Proper sequence generation (selection risk)	+	+	+	+	+	+	+	+	+
Selection hiding (selection bias)	+	+	+	+	+	+	+	+	+
Blinding of participants and staff (implementation bias)	+	+	+	+	+	+	+	+	+
Blinding of outcome evaluators (detection bias)	−	+	+	+	−	+	+	+	+
Incomplete results data (wear bias)	−	+	+	+	−	+	+	+	?
Selective reporting of results (notification bias)	+	+	+	+	+	+	+	+	+
Other sources of bias	?	?	?	?	?	?	?	?	?

Abbreviations: (+): low bias risk (−): high bias risk (?): unknown bias risk.

**Table 3:** Intervention characteristics.

Author	Year	Type of Intervention	Dosage	Intervention		Duration of the Intervention (Weeks)	Changes (Weeks)	Metabolomics	Metabolite Changes
Pietri et al. [44]	2019	Androgen deprivation therapy	DHEA 100 mg/day	GE = 12	GC = 6	8	8	Plasma	No changes observed
Ávila galvez A et al. [45]	2019	Capsules pomegranate, orange, lemon, olive, cocoa and grape seed extracts.	Three capsules daily	GE N = 19	GC N = 8	9	1–2	Urine, plasma, normal and malignant tissue	2,5-dihydroxybenzoic acid, 2,6-dihydroxybenzoic acid. urolithin-a 3-o-glucuronide
Chi et al. [46]	2020	Extreme low-carbohydrate diet (LCD) + Androgen deprivation therapy	Not specified	GE N = 19	GC N = 21	24	12–24	Plasma	Dihydroxycholestanoyl taurine, dodecanedioic acid, eicosatetraenoic acid, palmitoylcarnitine, oleoylcarnitine, 2-Aminoadipic acid, malonylcarnitine, octanoylarnitine, hexanoylcarnitine, myristoylcarnitine, decanoylcarnitine, heptanoycarnitine, dodecanoylcarnitine, androsterone sulfate, hydroxymyristoylcarnitine, palmitelaidic acid, 3-hydroxybutryc acid
Qu et al. [47]	2021	Neoadjuvant docetaxel + Androgen deprivation therapy	docetaxel (75 mg/m^2^) every 3 weeks	GE N = 12	GC N = 10	24	12–24	tumoral tissue	Citrate, succinic acid, glutamine, GSSG, adenine, glycerol 3-phosphate, PC, PE, LPE, GSH, PS, uridine
Lee et al. [48]	2021	Sodium Selenite Injection	500 µg sodium selenite, five times over 2 weeks.	GE N = 15	GC N = 14	2	2	Plasma	Cortisone, LTB4-DMA y PGE3, elevated in the experimental group
Febvey-Combes et al. [49]	2021	Aerobic exercise and dietary advice	exercise sessions 2–3 times a week supervised by a trainer.	GE N = 40	GC N = 18	24	No changes observed	Plasma	No changes observed
Hanada et al. [50]	2021	Herbal medicine Daikenchuto	DKT (5 g) orally three times daily	GE N = 8	GC N = 9	4	4	Plasma and faeces	Decrease in arachidonic acid, serratia and bilophila.
Zarei et al. [51]	2021	Dietary Navy Bean Intake	35 g of bean powder/day	GE:10	GC = 10	4	4	Plasma and urine	2,3-dihidroxi-2-metilbutirato S-methylcysteine, plasma pipecolate, urinary S-adenosylhomocysteine
Ávila-Galvez B et al. [52]	2021	Curcumin capsules	Three capsules/day (extracts of turmeric, red clover and flaxseed plus resveratrol; 296.4 mg phenolics/capsule; 296.4 mg phenolics/capsule)	GE N = 26	GC N = 13	From diagnosis to surgery	1–2	Plasma, urine, malignant tissue, normal tissue	4′-O-glucuronide, demethoxycurcumin curcumin, resveratrol 3-O-glucuronide, dihydroresveratrol 3-O-glucuronide, resveratrol 3-O-sulfate, and resveratrol 3-O-sulfate.

Abbreviations: GE: experimental group, GC: control group; DHEA: Dehydroepiandrosterone; mg: miligram; m^2^: square meter; GSSG: glutathione sulfide; PC: elevated phosphocholine; PE: Phosphatidylethanolamine; LPE: lysophosphatidylethanolamine; GSH: Glutation; PS: Phosphatidylserine; µg: microgram; LTB4: Leukotriene B4; DMA: Dodecanamide; PGE3: Prostaglandin E3; DKT: Daikenchuto; g: gram.

**Table 4:** Specific data in breast cancer and changes in the experimental group.

Author	Year	Cancer	Results of Oncometabolite
Pietri et al. [44]	2019	Breast Cancer	No significant changes in oncometabolites were observed.
Ávila Galvez et al. [45]	2019	Breast Cancer	Changes in: urolithin A-3-O-glucuronide, 2,5-dihydroxybenzoic acid and resvera-trol-3-O-sulfate (after surgery).
Lee et al. [48]	2021	Breast Cancer	Corticosterone, LTB4-DMA and PGE levels increased in the experimental group.
Febveys-Combes et al. [49]	2021	Breast Cancer	No significant differences were observed
Ávila Galvez et al. [52]	2021	Breast Cancer	Curcumin may offer long-term anticancer effects

Abbreviations: LTB4: Leukotriene B4; DMA: Dodecanamide; PGE: Prostaglandin.

**Table 5:** Specific data in prostate cancer and changes in the experimental group.

Author	Year	Cancer	Results of Oncometabolite
Chi et al. [46]	2020	Prostate Cancer	Decreased steroid synthesis, androgen levels, 3-hydroxybutyric acid, ketogenesis, acylcarnitines, and 3-formylindole
Qu et al. [47]	2021	Prostate Cancer	Beneficial changes in nucleotide synthesis, lipid, citric acid, and glutathione metabolism.

**Table 6:** Specific data in colon cancer (colorectal) and changes in the experimental group.

Author	Year	Cancer	Results of Oncometabolite
Hanada et al. [50]	2021	Colon Cancer	Decreased levels of plasma lipid mediators associated with the pro-inflammatory arachidonic acid cascade in the Daikenchuto group.
Zarei et al. [51]	2021	Colorectal Cancer	The following were increased in the experimental group: (i) 2,3-dihydroxy-2-methylbutyrate, (ii) S-methylcysteine and pipecolate in plasma and (iii) S-adenosylhomocysteine and cysteine in urine.

## Data Availability

All data associated with this study is present in the paper. All requests for other materials will be reviewed by the corresponding author to verify whether the request is subject to any intellectual property or confidentiality obligations. No protocol was prepared for this review.

## Supplementary Materials

The following supporting information can be downloaded at: https://www.mdpi.com/article/10.3390/cancers15174297/s1, Table S1: Search strategies; Table S2: GRADE System.

## References

[1] Salaverry O. (2013). La etimología del cáncer y su curioso curso histórico. Rev. Peru Med. Exp. Salud Publica.

[2] Halperin E.C. (2004). Paleo-oncology: The role of ancient remains in the study of cancer. Perspect. Biol. Med..

[3] Salas D., Peiró R. (2013). Evidencias sobre la prevención del cáncer [Evidence on the prevention of cancer]. Rev. Esp. Sanid Penit..

[4] Muhamad N., Na-Bangchang K. (2020). Metabolite Profiling in Anticancer Drug Development: A Systematic Review. Drug Des. Dev. Ther..

[5] Conceptos, Teorías y Factores Psicosociales en la Adaptación al Cáncer [Actas Esp. Psiquiatr. 2005]—Medes. https://medes.com/publication/19455.

[6] Gallo Cantafio M.E., Grillone K., Caracciolo D., Scionti F., Arbitrio M., Barbieri V., Pensabene L., Guzzi P.H., Di Martino M.T. (2018). From Single Level Analysis to Multi-Omics Integrative Approaches: A Powerful Strategy towards the Precision Oncology. High Throughput.

[7] Hobbins L., Gaoua N., Hunter S., Girard O. (2019). Psycho-physiological responses to perceptually-regulated interval runs in hypoxia and normoxia. Physiol. Behav..

[8] McTiernan A., Friedenreich C.M., Katzmarzyk P.T., Powell K.E., Macko R., Buchner D., Pescatello L.S., Bloodgood B., Tennant B., Vaux-Bjerke A. (2019). Physical Activity in Cancer Prevention and Survival: A Systematic Review. Med. Sci. Sports Exerc..

[9] Sung H., Ferlay J., Siegel R.L., Laversanne M., Soerjomataram I., Jemal A., Bray F. (2021). Global Cancer Statistics 2020: GLOBOCAN Estimates of Incidence and Mortality Worldwide for 36 Cancers in 185 Countries. CA Cancer J. Clin..

[10] Wishart D. (2022). Metabolomics and the Multi-Omics View of Cancer. Metabolites.

[11] Griffin J.L., Shockcor J.P. (2004). Metabolic profiles of cancer cells. Nat. Rev. Cancer.

[12] Schmidt D.R., Patel R., Kirsch D.G., Lewis C.A., Vander Heiden M.G., Locasale J.W. (2021). Metabolomics in cancer research and emerging applications in clinical oncology. CA Cancer J. Clin..

[13] Karlsson O., Rocklöv J., Lehoux A.P., Bergquist J., Rutgersson A., Blunt M.J., Birnbaum L.S. (2021). The human exposome and health in the Anthropocene. Int. J. Epidemiol..

[14] Aprile F., Bruno G., Palma R., Mascellino M.T., Panetta C., Scalese G., Oliva A., Severi C., Pontone S. (2021). Microbiota Alterations in Precancerous Colon Lesions: A Systematic Review. Cancers.

[15] Reglero C., Reglero G. (2019). Precision Nutrition and Cancer Relapse Prevention: A Systematic Literature Review. Nutrients.

[16] Koopaie M., Kolahdooz S., Fatahzadeh M., Manifar S. (2022). Salivary biomarkers in breast cancer diagnosis: A systematic review and diagnostic meta-analysis. Cancer Med..

[17] Leysen L., Lahousse A., Nijs J., Adriaenssens N., Mairesse O., Ivakhnov S., Bilterys T., Van Looveren E., Pas R., Beckwée D. (2019). Prevalence and risk factors of sleep disturbances in breast cancersurvivors: Systematic review and meta-analyses. Support Care Cancer.

[18] Takayama T., Tsutsui H., Shimizu I., Toyama T., Yoshimoto N., Endo Y., Inoue K., Todoroki K., Min J.Z., Mizuno H. (2016). Diagnostic approach to breast cancer patients based on target metabolomics in saliva by liquid chromatography with tandem mass spectrometry. Clin. Chim. Acta.

[19] Paz E.A., LaFleur B., Gerner E.W. (2014). Polyamines are oncometabolites that regulate the LIN28/let-7 pathway in colorectal cancer cells. Mol. Carcinog..

[20] Kdadra M., Höckner S., Leung H., Kremer W., Schiffer E. (2019). Metabolomics Biomarkers of Prostate Cancer: A Systematic Review. Diagnostics.

[21] Feng L.R., Wolff B.S., Lukkahatai N., Espina A., Saligan L.N. (2017). Exploratory Investigation of Early Biomarkers for Chronic Fatigue in Prostate Cancer Patients Following Radiation Therapy. Cancer Nurs..

[22] Czerwińska M., Bilewicz A., Kruszewski M., Wegierek-Ciuk A., Lankoff A. (2020). Targeted Radionuclide Therapy of Prostate Cancer-From Basic Research to Clinical Perspectives. Molecules.

[23] Wang L., Lu B., He M., Wang Y., Wang Z., Du L. (2022). Prostate Cancer Incidence and Mortality: Global Status and Temporal Trends in 89 Countries From 2000 to 2019. Front. Public Health.

[24] Wang G., Zhao D., Spring D.J., DePinho R.A. (2018). Genetics and biology of prostate cancer. Genes Dev..

[25] Zhang W., Zhang K. (2023). Quantifying the Contributions of Environmental Factors to Prostate Cancer and Detecting Risk-Related Diet Metrics and Racial Disparities. Cancer Inform..

[26] Glunde K., Bhujwalla Z.M., Ronen S.M. (2011). Choline metabolism in malignant transformation. Nat. Rev. Cancer.

[27] Lala P.K., Chakraborty C. (2001). Role of nitric oxide in carcinogenesis and tumour progression. Lancet Oncol..

[28] Mi S., Gong L., Sui Z. (2020). Friend or Foe? An Unrecognized Role of Uric Acid in Cancer Development and the Potential Anticancer Effects of Uric Acid-lowering Drugs. J. Cancer.

[29] Kukko V., Kaipia A., Talala K., Taari K., Tammela T.L.J., Auvinen A., Murtola T.J. (2022). Allopurinol and prostate cancer survival in a Finnish population-based cohort. Prostate Cancer Prostatic Dis..

[30] Ni Y., Xie G., Jia W. (2014). Metabonomics of human colorectal cancer: New approaches for early diagnosis and biomarker discovery. J. Proteome Res..

[31] Zwezerijnen-Jiwa F.H., Sivov H., Paizs P., Zafeiropoulou K., Kinross J. (2023). A systematic review of microbiome-derived biomarkers for early colorectal cancer detection. Neoplasia.

[32] Mallafré-Muro C., Llambrich M., Cumeras R., Pardo A., Brezmes J., Marco S., Gumà J. (2021). Comprehensive Volatilome and Metabolome Signatures of Colorectal Cancer in Urine: A Systematic Review and Meta-Analysis. Cancers.

[33] Wanders D., Hobson K., Ji X. (2020). Methionine Restriction and Cancer Biology. Nutrients.

[34] Zhang H.-L., Zhang A.-H., Miao J.-H., Sun H., Yan G.-L., Wu F.-F., Wang X.-J. (2019). Targeting regulation of tryptophan metabolism for colorectal cancer therapy: A systematic review. RSC Adv..

[35] Junn E., Mouradian M.M. (2012). MicroRNAs in neurodegenerative diseases and their therapeutic potential. Pharmacol. Ther..

[36] Luszczynska A., Benight C.C., Cieslak R. (2009). Self-Efficacy and Health-Related Outcomes of Collective Trauma. Eur. Psychologist..

[37] Tian J., Xue W., Yin H., Zhang N., Zhou J., Long Z., Wu C., Liang Z., Xie K., Li S. (2020). Differential Metabolic Alterations and Biomarkers Between Gastric Cancer and Colorectal Cancer: A Systematic Review and Meta-Analysis. Onco. Targets Ther..

[38] Yang L., Wang Y., Cai H., Wang S., Shen Y., Ke C. (2020). Application of metabolomics in the diagnosis of breast cancer: A systematic review. J. Cancer.

[39] Lima A.R., Pinto J., Amaro F., Bastos M.L., Carvalho M., Guedes de Pinho P. (2021). Advances and Perspectives in Prostate Cancer Biomarker Discovery in the Last 5 Years through Tissue and Urine Metabolomics. Metabolites.

[40] Welch V., Petticrew M., Tugwell P., Moher D., O’Neill J., Waters E., White H. (2012). PRISMA-Equity 2012 extension: Reporting guidelines for systematic reviews with a focus on health equity. PLoS Med..

[41] Rayyan—A Web and Mobile App for Systematic Reviews|Systematic Reviews|Full Text. https://systematicreviewsjournal.biomedcentral.com/articles/10.1186/s13643-016-0384-4.

[42] Cochrane Handbook for Systematic Reviews of Interventions. https://handbook-5-1.cochrane.org/.

[43] Aguayo-Albasini J.L., Flores-Pastor B., Soria-Aledo V. (2014). Sistema GRADE: Clasificación de la calidad de la evidencia y graduación de la fuerza de la recomendación [GRADE system: Classification of quality of evidence and strength of recommendation]. Cir. Esp..

[44] Pietri E., Massa I., Bravaccini S., Ravaioli S., Tumedei M.M., Petracci E., Donati C., Schirone A., Piacentini F., Gianni L. (2019). Phase II Study of Dehydroepiandrosterone in Androgen Receptor-Positive Metastatic Breast Cancer. Oncologist.

[45] Ávila-Gálvez M.Á., García-Villalba R., Martínez-Díaz F., Ocaña-Castillo B., Monedero-Saiz T., Torrecillas-Sánchez A., Abellán B., González-Sarrías A., Espín J.C. (2019). Metabolic Profiling of Dietary Polyphenols and Methylxanthines in Normal and Malignant Mammary Tissues from Breast Cancer Patients. Mol. Nutr. Food Res..

[46] Chi J., Lin P., Tolstikov V., Oyekunle T., Chen E.Y., Bussberg V., Greenwood B., Sarangarajan R., Narain N.R., Kiebish M.A. (2020). Metabolomic effects of androgen deprivation therapy treatment for prostate cancer. Cancer Med..

[47] Qu F., Gu Y., Xue M., He M., Zhou F., Wang G., Peng Y. (2021). Impact of therapy on cancer metabolism in high-risk localized prostate cancer treated with neoadjuvant docetaxel and androgen deprivation therapy. Prostate.

[48] Lee H., Lee B., Kim Y., Min S., Yang E., Lee S. (2021). Effects of Sodium Selenite Injection on Serum Metabolic Profiles in Women Diagnosed with Breast Cancer-Related Lymphedema-Secondary Analysis of a Randomized Placebo-Controlled Trial Using Global Metabolomics. Nutrients.

[49] Febvey-Combes O., Jobard E., Rossary A., Pialoux V., Foucaut A.-M., Morelle M., Delrieu L., Martin A., Caldefie-Chézet F., Touillaud M. (2021). Effects of an Exercise and Nutritional Intervention on Circulating Biomarkers and Metabolomic Profiling During Adjuvant Treatment for Localized Breast Cancer: Results from the PASAPAS Feasibility Randomized Controlled Trial. Integr. Cancer Ther..

[50] Hanada K., Wada T., Kawada K., Hoshino N., Okamoto M., Hirata W., Mizuno R., Itatani Y., Inamoto S., Takahashi R. (2021). Effect of herbal medicine daikenchuto on gastrointestinal symptoms following laparoscopic colectomy in patients with colon cancer: A prospective randomized study. Biomed Pharmacother..

[51] Zarei I., Baxter B.A., Oppel R.C., Borresen E.C., Brown R.J., Ryan E.P. (2021). Plasma and Urine Metabolite Profiles Impacted by Increased Dietary Navy Bean Intake in Colorectal Cancer Survivors: A Randomized-Controlled Trial. Cancer Prev. Res..

[52] Ávila-Gálvez M., González-Sarrías A., Martínez-Díaz F., Abellán B., Martínez-Torrano A.J., Fernández-López A.J., Giménez-Bastida J.A., Espín J.C. (2021). Disposition of Dietary Polyphenols in Breast Cancer Patients’ Tumors, and Their Associated Anticancer Activity: The Particular Case of Curcumin. Mol. Nutr. Food Res..

[53] Barcenas C., Hurvitz S., Di Palma J., Bose R., Chien A., Iannotti N., Marx G., Brufsky A., Litvak A., Ibrahim E. (2020). Improved tolerability of neratinib in patients with HER2-positive early-stage breast cancer: The CONTROL trial. Ann. Oncol..

[54] Tutt A.N., Garber J.E., Kaufman B., Viale G., Fumagalli D., Rastogi P., Gelber R.D., de Azambuja E., Fielding A., Balmaña J. (2021). Adjuvant Olaparib for Patients with BRCA1- or BRCA2-Mutated Breast Cancer. N. Engl. J. Med..

[55] Akinyele O., Wallace H.M. (2022). Understanding the Polyamine and mTOR Pathway Interaction in Breast Cancer Cell Growth. Med. Sci..

[56] Razavi M., Jamilian M., Kashan Z.F., Heidar Z., Mohseni M., Ghandi Y., Bagherian T., Asemi Z. (2016). Selenium Supplementation and the Effects on Reproductive Outcomes, Biomarkers of Inflammation, and Oxidative Stress in Women with Polycystic Ovary Syndrome. Horm. Metab. Res..

[57] Kunihiro A.G., Brickey J.A., Frye J.B., Cheng J.N., Luis P.B., Schneider C., Funk J.L. (2022). Curcumin Inhibition of TGFβ signaling in bone metastatic breast cancer cells and the possible role of oxidative metabolites. J. Nutr. Biochem..

[58] Bahrami A., Atkin S.L., Majeed M., Sahebkar A. (2018). Effects of curcumin on hypoxia-inducible factor as a new therapeutic target. Pharmacol. Res..

[59] James M.I., Iwuji C., Irving G., Karmokar A., Higgins J.A., Griffin-Teal N., Thomas A., Greaves P., Cai H., Patel S.R. (2015). Curcumin inhibits cancer stem cell phenotypes in ex vivo models of colorectal liver metastases, and is clinically safe and tolerable in combination with FOLFOX chemotherapy. Cancer Lett..

[60] Keenan M.M., Chi J.T. (2015). Alternative fuels for cancer cells. Cancer J..

[61] Schmidt M.E., Meynköhn A., Habermann N., Wiskemann J., Oelmann J., Hof H., Wessels S., Klassen O., Debus J., Potthoff K. (2016). Resistance Exercise and Inflammation in Breast Cancer Patients Undergoing Adjuvant Radiation Therapy: Mediation Analysis from a Randomized, Controlled Intervention Trial. Int. J. Radiat. Oncol. Biol. Phys..

[62] Bye A., Sandmael J.A., Stene G.B., Thorsen L., Balstad T.R., Solheim T.S., Pripp A.H., Oldervoll L.M. (2020). Exercise and Nutrition Interventions in Patients with Head and Neck Cancer during Curative Treatment: A Systematic Review and Meta-Analysis. Nutrients.

[63] Wang L., Wu L., Qian C., Ju Y., Liu T., Chen Y., Wang X. (2022). The Beneficial Effect of a Healthy Dietary Pattern on Androgen Deprivation Therapy-Related Metabolic Abnormalities in Patients with Prostate Cancer: A Meta-Analysis Based on Randomized Controlled Trials and Systematic Review. Metabolites.

[64] Ben Zichri S., Kolusheva S., Shames A.I., Schneiderman E.A., Poggio J.L., Stein D.E., Doubijensky E., Levy D., Orynbayeva Z., Jelinek R. (2021). Mitochondria membrane transformations in colon and prostate cancer and their biological implications. Biochim. Biophys. Acta Biomembr..

[65] Hoshino N., Kawada K., Hida K., Wada T., Takahashi R., Yoshitomi M., Sakai Y. (2017). Effect of Daikenchuto (TJ-100) on gastrointestinal symptoms following laparoscopic colectomy in patients with colon cancer: Study protocol for a randomized controlled trial. Trials.

[66] Han S., Gao J., Zhou Q., Liu S., Wen C., Yang X. (2018). Role of intestinal flora in colorectal cancer from the metabolite perspective: A systematic review. Cancer Manag. Res..

[67] Bischoff-Ferrari H.A., Willett W.C., Manson J.E., Dawson-Hughes B., Manz M.G., Theiler R., Braendle K., Vellas B., Rizzoli R., Kressig R.W. (2022). Combined Vitamin D, Omega-3 Fatty Acids, and a Simple Home Exercise Program May Reduce Cancer Risk Among Active Adults Aged 70 and Older: A Randomized Clinical Trial. Front. Aging.

