# Supplementary material for: Effectiveness of Treatments That Alter Metabolomics in Cancer Patients—A Systematic Review
Source: Cancers (Basel). 2023 Aug 28;15(17):4297. doi: 10.3390/cancers15174297 (PMC10486463; doi:10.3390/cancers15174297)
Supplement: Supplementary file 1 [file cancers-15-04297-s001.zip › cancers-2453594-supplementary.pdf]

Supplementary Tables

- Table S1- Search strategies
- Table S2. GRADE System

Table S1. Search strategies

Platform	Search Strategies
PubMed	((“Neoplasms”[Mesh]) AND (“metabolomics”[Mesh] OR “metabolome”[Mesh] OR “genetics”[Mesh] OR “mycobiome”[Mesh] OR “microbiota”[Mesh]) OR (“cancer pain”[Mesh] OR “pain”[Mesh] OR “quality of life”[Mesh]) OR (“Cancer”[tw] OR “neoplasms”[tw]) AND (“metabolomics”[tw] OR “metabolites”[tw] OR “metabolome”[tw] OR “genetics”[tw] OR “human genetics”[tw]) AND (“cancer pain”[tw] OR “pain”[tw] OR “quality of life”[tw]))
SCOPUSS	((“Cancer” OR “neoplasms”) AND (“metabolomics” OR “metabolites” OR “metabolome” OR “genetics” OR “human genetics”) AND (“cancer pain” OR “pain” OR “quality of life”)) OR ((“Neoplasms”) AND (“metabolomics” OR “metabolome” OR “genetics” OR “mycobiome” OR “microbiota”) OR (“cancer pain” OR “pain” OR “quality of life”))

LILACS<br>IBECS<br>(BVS)	Tw:((“Cancer” OR “neoplasms”) AND (“metabolomics” OR “metabolites” OR “metabolome” OR “genetics” OR “human genetics”) AND (“cancer pain” OR “pain” OR “quality of life”)) OR Mh: ((“Neoplasms”) AND (“metabolomics” OR “metabolome” OR “genetics” OR “mycobiome” OR “microbiota”) OR (“cancer pain” OR “pain” OR “quality of life”))
Cochrane Library	#1 Cancer<br>#2 neoplasms<br>#3 metabolomics<br>#4 metabolites<br>#5 metabolome<br>#6 genetics<br>#7 human genetics<br>#8 cancer pain<br>#9 pain<br>#10 quality of life<br>#11 MeSH descriptor: [Neoplasms] explode all trees

	#12	MeSH descriptor: [Metabolomics] explode all trees
	#13	MeSH descriptor: [Metabolomics] explode all trees
	#14	MeSH descriptor: [Genetics] explode all trees
	#15	MeSH descriptor: [Mycobiome] explode all treeS
	#16	MeSH descriptor: [Microbiota] explode all trees
	#17	MeSH descriptor: [Cancer Pain] explode all trees
	#18	MeSH descriptor: [Pain] explode all treeS
	#19	MeSH descriptor: [Quality of Life] explode all trees
	#20	((#1 OR #2) AND (#3 OR #4 OR #5 OR #6 OR #7) AND (#8 OR #9 OR #10)) OR ((#11) AND (#12 OR #13 OR #14 OR #15 OR #16) AND (#17 OR #18 OR #19))
EBSCO	MH((“Neoplasms”) AND (“metabolomics” OR “metabolome” OR “genetics” OR “mycobiome” OR “microbiota”) OR (“cancer pain” OR “pain” OR “quality of life”)) OR ((“Cancer” OR “neoplasms”) AND (“metabolomics” OR “metabolites” OR “metabolome” OR “genetics” OR “human genetics”) AND (“cancer pain” OR “pain” OR “quality of life”))	
Web of science	TS=((“Cancer” OR “neoplasms”) AND (“metabolomics” OR “metabolites” OR “metabolome” OR “genetics” OR “human genetics”) AND (“cancer pain” OR “pain” OR “quality of life”))	

Table S2. GRADE System

Author(s): Dina Hamed-Hamed, Santiago Navarro-Ledesma

Question: Metabolomics compared to for cancer patients

Setting: University of Granada

Bibliography:

Certainty assessment							Impact	Certainty	Importance
№ of studies	Study design	Risk of bias	Inconsistency	Indirectness	Imprecision	Other considerations			
**Metabolomics (follow-up: median 8 weeks)**									
9	randomised trials	not serious	serious[a]	not serious	serious[b]	dose response gradient	Of the 3 studies that recedived pharmacological tratment, one found no difference in the metabolome of the patients, the other two studies did. The study using exercise as an intervention also showed no change in metabolomics. There were very beneficial changes in metabolites in the 4 studies that used dietary supplements and the study that used traditional Chineses medicine. [c]	⊕⊕⊕◯ Moderate	NOT IMPORTANT

CI: confidence interval

Explanations

a. Interventions have been shown to improve the human metabolome, but specific biomarkers have yet to be studied to eradicate the disease

b. Due to the low sample size of many studies

c. Of the 3 studies that received pharmacological treatment, one found no difference in the metabolome of the patients, the other two studies did.The study using exercise as an intervention also showed no change in metabolomics. There were very beneficial changes in metabolites in the 4 studies that used dietary supplements and the study that used traditional Chinese medicine.
